# “Entrepreneurship” from the lens of enlightenment: Impacts of religiosity and spiritual intelligence on social entrepreneurial intentions

**DOI:** 10.1371/journal.pone.0285140

**Published:** 2023-10-10

**Authors:** Hongxia Jia, Shahid Iqbal, Arslan Ayub

**Affiliations:** 1 Department of Teaching and Research, Hebei Polytechnic Institute, Shijiazhuang, China; 2 Management Studies Department, Bahria University, Islamabad, Pakistan; 3 National School of Management Studies, The University of Faisalabad, Punjab, Pakistan; Tallinn University: Tallinna Ulikool, ESTONIA

## Abstract

Despite an escalated gravity of interest in exploring factors that shape university students’ social entrepreneurial intentions, there are significant gaps in our understanding of this phenomenon. The current study examines the boundary effects of religiosity and spiritual intelligence to predict university students’ social entrepreneurial intentions. The authors collected the data from university students in their final years in multiple waves and employed SmartPLS (v 4.0) for data analysis. Our findings indicate that religiosity can affect social entrepreneurial intentions through two paths: intrinsic motivation mediates the association between intrinsic religiosity and social entrepreneurial intentions, and extrinsic motivation mediates the relationship between extrinsic religiosity and social entrepreneurial intentions. Further, spiritual intelligence moderates the relationship between intrinsic religiosity and social entrepreneurial intentions, mediated by intrinsic motivation such that at high levels of spiritual intelligence the association is more potent and vice versa. This is the first study that examines the boundary conditions of social entrepreneurial intentions of university students by employing the lens of religiosity and spirituality. The paper presents substantial theoretical and practical implications.

## Introduction

*“Just as a candle cannot burn without fire*, *man cannot live without a spiritual life” (Buddha)*. A life full of meaning with the purpose of serving humanity has seized the attention of researchers and practitioners for the past few decades. Particularly in the business parlance, one way of putting it into practice is by translating the societal aspects into entrepreneurship, *i*.*e*., social entrepreneurship [1–3]. Social enterprises and entrepreneurs participating in economic and social developments have recently attracted scholarly attention [4–6]. Social entrepreneurship includes efforts aimed at addressing some of the urgent “social needs” and refers to “the practice of addressing social problems by means of markets” [7, p 333]. By the same token, Dees [8] described social entrepreneurs as “social agents that engage in a process of continuous innovation, adaptation and learning and that exhibit a high sense of accountability to their constituencies and the outcomes they create”. Social entrepreneurship can take place within and across “nonprofit”, “business”, and “public” sectors [9]. It stems from business logic that embarks upon unique and entrepreneurial ways to promote the societal interests of marginalized, excluded or suffering segments that cannot change their situation [10].

Considering the importance of social entrepreneurship in promoting economic and societal impacts, there is an escalated gravity of interest in understanding the drivers of social entrepreneurial intentions to provide a finer-grain understanding of the phenomenon. Social entrepreneurial intentions refer to the determination, desire, or belief to commence a new social enterprise in the future [11]. In this milieu, a growing stream of research has sought to examine the antecedents of social entrepreneurial intentions in recent years [1, 5, 12]. Remarkably, there is a burgeoning emphasis on university students pursuing entrepreneurial careers guided by a social mission [2, 11]. Theoretical frameworks encapsulating university students’ social entrepreneurial intentions are compelling to study for several reasons. First, entrepreneurial careers provide an alternative to employment for oneself, combating unemployment [13]. Second, it leverages job creation and stimulates economic growth [14]. Third, social entrepreneurship can positively impact a wide range of sectors, including education, environmental protection, and healthcare, among others [3, 4, 6]. Not surprisingly, research studies investigating the determinants of social entrepreneurial intentions have become the “hotspot” research focus in the entrepreneurship domain.

Despite an escalated gravity of interest in exploring factors that shape university students’ social entrepreneurial intentions, there are significant gaps in our understanding of this phenomenon. First, relatively little is known about the factors that provoke individuals’ desire to establish a social enterprise [13]. Although it has been theorized that religiosity can be associated with pro-sociality, few studies have empirically investigated these relationships [15] (The authors used religious parables as narratives that elicit and encourage ethical discussions in social entrepreneurship courses.). We address this gap by assessing religiosity as an antecedent of social entrepreneurial intentions. The religiosity orientation is categorized into two dimensions, *i*.*e*., “intrinsic religiosity” (*i*.*e*., internalization of religious values) and “extrinsic religiosity” (*i*.*e*., utilitarian and instrumental dimensions of religion) [16]. We predict that religiosity, *i*.*e*., intrinsic religiosity and extrinsic religiosity, is a crucial factor in determining university students’ social entrepreneurial intentions. Exploring religiosity in this context is essential because substantial evidence supports our theoretical deduction. For instance, religiosity has been linked with ethical behavior, values [17], and pro-sociality [18]. Therefore, extending the implications of religiosity in the social entrepreneurship domain is novel and pertinent. Besides, a review of literature on the exchange discussion between Galen [19], who claims that "the proposed link between religiosity and pro-sociality is a fallacy", and Myers [20], who reinforces a positive association between religiosity and pro-sociality, warrants further empirical security of this phenomenon [21].

Furthermore, the current study advances the hypothesized relationship by predicting a causal mechanism, *i*.*e*., intrinsic motivation and extrinsic motivation, through which intrinsic religiosity and extrinsic religiosity translate into social entrepreneurial intentions. The study anchors on the self-determination theory (SDT) [22, 23] and projects two paths through which religiosity fosters social entrepreneurial intentions. The study proposes that intrinsic religiosity augments social entrepreneurial intentions through intrinsic motivation, and extrinsic religiosity promotes social entrepreneurial intentions through extrinsic motivation. To the best of the authors’ knowledge, no empirical studies have tested these relationships. By addressing this gap, our study aims to provide a more nuanced understanding of the implications of religiosity for social entrepreneurial intentions, which can help educationists and policymakers leverage this phenomenon more effectively.

**Fig 1:**
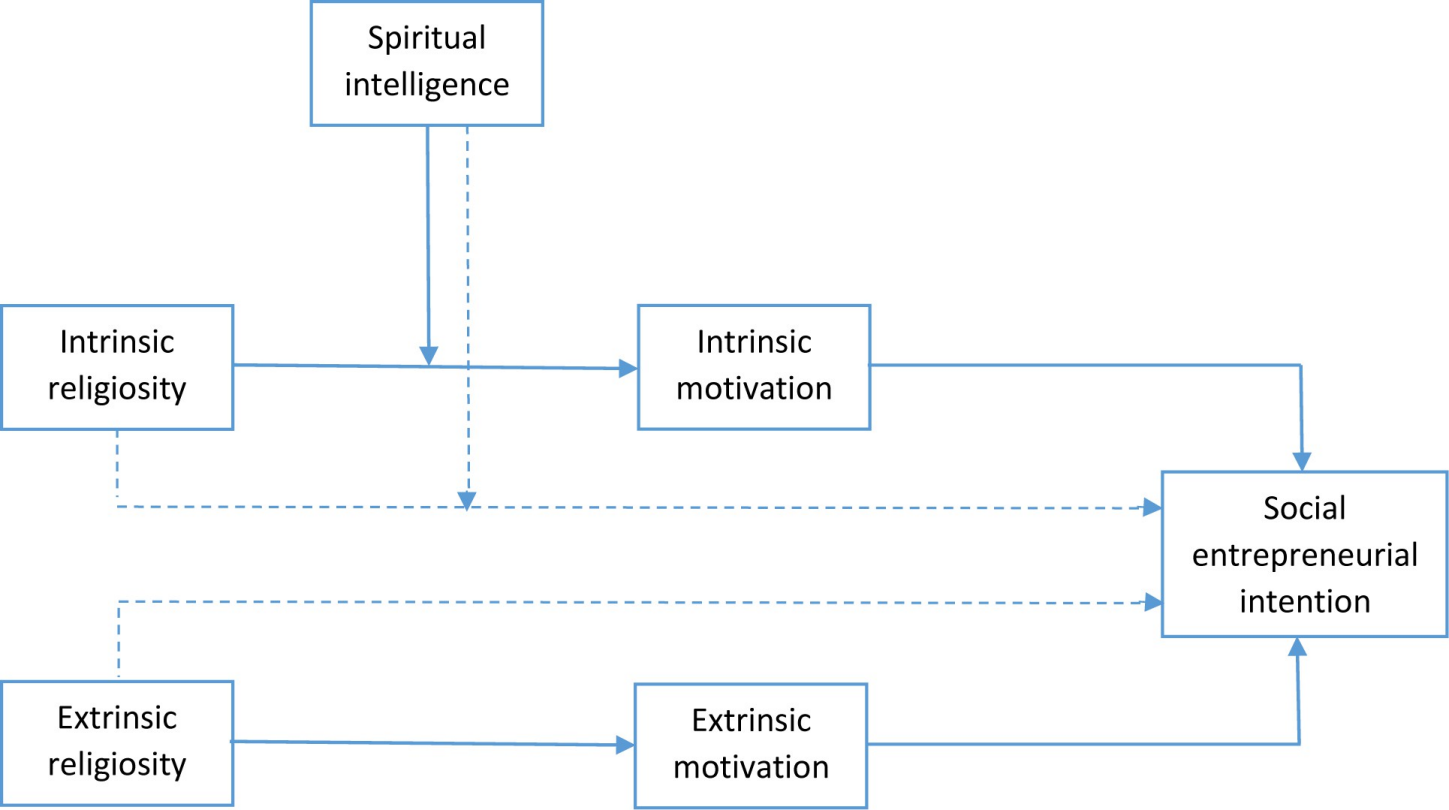
Conceptual model.

Last but not least, our study extends the boundary conditions of religiosity and social entrepreneurial intentions by proposing the moderator effect of spiritual intelligence between intrinsic religiosity and social entrepreneurial intentions. According to King [24], spiritual intelligence reflects an individual’s mental capacities that leverage the mastery of spiritual values, recognition of a transcendent self, enhancement of meaning, and deep existential reflection through broadening awareness, integration and adaptive application of transcendental and nonmaterial aspects of one’s existence. It offers spiritual insights that enable individuals to direct their attention and actions to a more significant cause based on spiritual values [25]. Drawing on the self-efficacy theory, individuals hold beliefs that they are capable of influencing the environment through their actions and behaviors. The central premise of spiritual intelligence is one’s recognition of his/her deep self to well-being and for fulfilling life [26]. Social scholars have linked spiritual intelligence with improved self-efficacy and higher levels of social responsibility [25, 27, 28]. Therefore, it offers a maximum explanation to reinforce the association between intrinsic religiosity and social entrepreneurial intentions. Several prior studies have assessed the moderating role of spiritual intelligence in the work context [29] and entrepreneurship domains [30]. We specifically propose that (1) intrinsic motivation mediates the association between intrinsic religiosity and social entrepreneurial intentions, (2) extrinsic motivation mediates the association between extrinsic religiosity and social entrepreneurial intentions, and (3) spiritual intelligence moderates the relationship between intrinsic religiosity and social entrepreneurial intentions through the mediating role of intrinsic motivation (Fig 1).

## Theoretical framework

### Religiosity and social entrepreneurship

Religiosity is defined as “an organized set of beliefs pertaining to higher power, supported by rituals, practices and a shared code of ethics” [31]. Researchers argue that to be religious, individuals need to hold religious beliefs and embed them in their day-to-day life experiences [21]. There has been a growing scholarly debate on the link between religiosity and entrepreneurship in recent years [32]. For instance, Judge and Douglas [33, p52] conducted a longitudinal study on US-based entrepreneurs. They found religiosity "to be instrumental in assisting the founders [to] cope with limited resources, overcome seemingly insurmountable obstacles and identify new business opportunities". The notion is that one’s religious orientation, engendering them to face the world’s trials by eliciting hope, enables entrepreneurs to face uncertainties linked with the start-up process [34]. Individuals with a religious orientation can deal with challenges that might come the way of entrepreneurs.

Moreover, prior religious works have linked religion with ethical decision-making and ethical work in organizations [17, 35]. In a similar way, it is argued that religious affiliations enhance altruistic and prosocial behaviors, facilitating individuals to establish a social enterprise by pursuing sustainable and social business practices [36]. Thus, religion is expected to translate into an entrepreneur’s mission to provide financial gains to society. As Judge and Douglas [33, p. 58] substantiated that religion fosters prosocial behaviors, *i*.*e*., “seeking to make life a little better for everyone whose path [the entrepreneur] crossed [and]…to fulfil the expectations of [the] community”. Another argument supporting this corollary portrays a link between religion and social trust in building social capital [37]. For instance, entrepreneurs who reflect their social identity based on actual or claimed religious convictions are more likely to gain the trust of business partners, customers, and investors and may exaggerate the resource flows [38]. On the contrary, several preliminary studies have also reported that the link between religion and entrepreneurship is not always unidirectional [37], or in some cases, is nonexistent (see, for example, Audretsch *et al*. [39]. This makes our study all the more salient to contribute to the existing empirical research on the subject by investigating the link between religiosity and social entrepreneurial orientations.

### Self-determination theory

Motivation is crucial in determining individuals’ actions and behaviors and has substantial implications for understanding intentions [40]. Prior studies to understand individual and organizational behavior have extensively utilized the construct due to its enormous standing in the context [41]. Similarly, motivation is relevant to understanding factors that fuel the creation of new projects [42]. As validated by Cnossen *et al*. [43], motivation engenders individuals to govern the direction and intensity of their actions. In this regard, individual’s perception of the feasibility and value of an endeavor determines their action and effort to achieve a goal.

According to Deci and Ryan [22]; Ryan and Deci [23], motivation is embedded in the SDT, which divides it into “autonomous” and “controlled”. Autonomous motivation reflects an individual’s involvement or desire to engage in an activity based on the inherent pleasure in partaking in the activity. In comparison, controlled motivation represents actions and behaviors driven by external factors, *e*.*g*., a sense of duty and obligation or external rewards. Drawing on the SDT, our projections are hypothesized as individuals who internalize religious values, *e*.*g*., pro-sociality, are energetic and self-driven, and perform actions irrespective of the external rewards that yield inner satisfaction and a sense of fulfilment, *i*.*e*., social entrepreneurial intentions. On the contrary, individuals who view religion’s utilitarian and materialistic aspects are guided by external factors, *e*.*g*., social recognition or monetary payoff, and direct their behavior towards goal attainment, *i*.*e*., social entrepreneurial intentions. Although intrinsic and extrinsic motivation are opposite to each other, the proponents of motivation endorse that both types of incentives may be complementary and reinforce each other [44]. For instance, Amabile [45] studied the roles of both types of motivation on entrepreneurial behaviors and found that the synergy between intrinsic and extrinsic motivation enhances entrepreneurial creativity, wherein intrinsic motivation plays a stronger role. Hence, our predictions are amendable to empirical tests of the associations between religiosity (*i*.*e*., intrinsic and extrinsic religiosity), motivation (*i*.*e*., intrinsic and extrinsic motivation), and social entrepreneurial intentions.

## Hypotheses

### The mediating role of motivation

The current study proposes that religiosity impacts individuals’ social entrepreneurial intentions through motivation. According to Allport and Ross [16], whether religiosity holds positive or negative implications for ethical issues and pro-sociality is determined by an individual’s religious orientation. According to the authors, religious orientations are of two types, *i*.*e*., “intrinsic religiosity” and “extrinsic religiosity” [16]. Intrinsic religiosity is "a motivation driven by the core values of religion—individuals with an intrinsic religious orientation endeavor to reflect the true spirit of their religious beliefs in their actions”. On the contrary, extrinsic religiosity is described as “a religious motivation driven by personal benefit—religion is considered as a means to some form of utility, either personal or social (e.g., joining a church to make business or social connections)”. Intrinsic religiosity has been described as just good, interiorized, and genuine, whereas extrinsic religiosity has often been presented as dogmatic, instrumental, self-serving, and egoistic [37].

We draw on the SDT and predict the link between religiosity and motivation. According to SDT, intrinsically religious individuals have an intrinsic religious motivation, and they embark upon "internalized regulation", that is fully acknowledged and assimilated into one’s value system. The engagement or desire to engage in the activity emanates from inherent enjoyment and interest in it and "for their own sake" [23]. A host of researchers in the recent past have identified the direct link between intrinsic religiosity and intrinsic motivation in the work context [46]. The authors sanctioned that intrinsic religiosity embraces “fundamental” values of religion, which enable individuals to reach the desired outcomes through fostering intrinsic motivation.

On the contrary, the SDT purports that extrinsic religiosity espouses extrinsic motivation, representing "behaviors done for reasons other than inherent satisfaction" [23]. According to the SDT [47], the extrinsic motivation continuum comprises controlled and autonomous orientations *namely* “external regulation”, “introjected regulation”, “identified regulation”, and “integrated regulation”. External regulation concerns behaviors that are externally driven and are based on external rewards or punishments. Introjected regulation represents “partial internalization”, in which behaviors are driven by internal rewards of “self-esteem” for success and by circumvention of guilt, shame, or anxiety for failure [48]. In juxtaposition, internalized regulation is autonomous in extrinsic motivation through "identified regulation" and "integrated regulation". Identified regulation indicates an individual’s will or volitional involvement in the activity because of the value ascertained to it, and the person consciously recognises or endorses it. In a similar vein, integrated regulation further extends the autonomous continuum of an individual’s extrinsic motivation by recognising the activity’s value and finding it to be harmonized with one’s core interests and values [47]. This supports our theoretical deduction that individuals who practice religion from instrumental and materialistic perspectives, *i*.*e*., extrinsic religiosity, are guided by the extrinsic motivation to reach the desired outcomes because they find external factors congruent with their core self-interests and values. Therefore,

*H1a. Intrinsic religiosity positively influences intrinsic motivation*.

*H1b. Extrinsic religiosity positively influences extrinsic motivation*.

Subsequently, we propose that motivation, *i*.*e*., intrinsic and extrinsic motivation, predicts individuals’ social entrepreneurial intentions. Social entrepreneurship predominantly addresses societal issues [49]; therefore, understanding motivations that shape intentions to establish social enterprises are a crucial concept in social entrepreneurship studies. The SDT proposes that individuals’ actions and behaviors are influenced by their orientation toward intrinsic and extrinsic motivation [23]. For instance, university students in their final years are more concerned about their career choices [42]. Individuals who choose to be social entrepreneurs may develop higher social entrepreneurial intentions guided by either intrinsic or extrinsic motivation to serve society. Individuals with intrinsic motivation orientation towards pro-sociality are more inclined in establishing a social enterprise because they find interest and pleasure in it, which yields inherent satisfaction in addressing societal problems through their business offerings. Furthermore, intrinsic motivation is linked with enhanced mastery experiences because it allows individuals to develop new skill sets as they are deeply involved in those activities [23]. Ultimately, pursuing entrepreneurial careers guided by intrinsic motivation may appear more beguiling to university students [14]. Further, Yamini [12] argued that intrinsic motivation for social entrepreneurship increases when people have a deeper affiliation for and interest in societal issues, which they find enjoyable, engaging, and positively challenging. Besides, a growing body of empirical evidence supports the promising role of intrinsic motivation in elevating performance outcomes, both in the occupational [50] and entrepreneurship contexts [12]. Individuals driven by intrinsic motivation to address societal issues are more likely to nurture social entrepreneurial intentions.

The study also predicts the positive correlation between extrinsic motivation and social entrepreneurial intentions. According to the SDT, individuals who intend to accomplish extrinsic goals (*e*.*g*., physical appearance, financial success, or fame) are extrinsically motivated to involve in any behavior [23]. Extrinsic motivation becomes more compelling for individuals choosing an entrepreneurial career [51], particularly social entrepreneurship, for individuals who aim to become famous and attractive in society [52]. Individuals successfully launching social enterprises become success stories in the media [53], and they are invited as guest speakers to conferences, workshops, and seminars [51]. They are perceived as providers of unique offerings and appear as heroes in society [54]. Furthermore, financial success has been reported to be another critical factor that entices individuals to pursue social entrepreneurial careers. Tan *et al*. [13] corroborated that social entrepreneurship promotes social success, which elevates the company’s financial success. As endorsed by Gali *et al*. [55, p. 29], in simple words, “success drives success”. Therefore, people more concerned with external goals are more likely to internalize their goals into actions; hence, they may develop a greater tendency toward social entrepreneurship. Thus,

*H2a. Intrinsic motivation positively influences social entrepreneurial intentions*.

*H2b. Extrinsic motivation positively influences social entrepreneurial intentions*.

In combination, anchored on the SDT, we propose that religiosity affects social entrepreneurial intentions through two paths. Individuals who espouse fundamental religious values, *i*.*e*., intrinsic religiosity, culminate into intrinsic motivation, promoting social entrepreneurial intentions. For them, demonstrating behaviors that enhance pro-sociality and altruism reap inherent satisfaction; they are intrinsically motivated to start a social enterprise. Similarly, people more concerned with external goals, *i*.*e*., fame, recognition, or monetary success, are more likely to ripen social entrepreneurial intentions through extrinsic motivation. In a nutshell, intrinsic motivation offers justifications for internal factors, while extrinsic motivation expounds external factors that might internalize religious values into one’s actions and behaviors and translate intrinsic and extrinsic religiosity into social entrepreneurial intentions.

*H3a. Intrinsic motivation mediates the association between intrinsic religiosity and social entrepreneurial intentions*.

*H3b. Extrinsic motivation mediates the association between extrinsic religiosity and social entrepreneurial intentions*.

### Moderator effect of spiritual intelligence

Although we expect a positive link between religiosity, *i*.*e*., intrinsic religiosity and extrinsic religiosity, and motivation, *i*.*e*., intrinsic motivation and extrinsic motivation (and social entrepreneurial intentions), the variability observed between the antecedents of social entrepreneurial intentions [5] suggests the potential for moderators. We, therefore, propose the moderating role of spiritual intelligence in the association between intrinsic religiosity and intrinsic motivation (and social entrepreneurial intentions). Since extrinsic religiosity reflects dogmatic, instrumental, and self-serving motives, manifesting utilitarian and instrumental dimensions of religion [16], we contemplate that spiritual intelligence will be less likely reinforcing the association between extrinsic religiosity and extrinsic motivation. In the similar thread, Ip *et al*. [56] analyzed the influence of extrinsic rewards between outcome expectations and social entrepreneurial intentions. The authors found that individuals with a higher tendency for the extrinsic rewards are less likely to manifest behaviors such as social entrepreneurial intentions. On contrary, individuals with a strong desire for intrinsic values and goals will be in a state of harmony with the nature of social enterprises. Hence, we expect the moderating role of spiritual intelligence in the relationship between intrinsic religiosity and social entrepreneurial intentions through intrinsic motivation. Zohar [57] defined spiritual intelligence as “a kind of intelligence that can solve the semantic and value issues, intelligence which can make the daily life activities richer in context, wider and more meaningful”. Spiritual intelligence allows individuals to employ spiritual values that are directed to elevate an individual’s performance [25]. Spiritual intelligence constitutes spirituality and intelligence conjoining into a single concept [58]. According to King and DeCicco [59], there are four components of spiritual intelligence:

1. *Critical existential thinking–“Ability to think critically about the truth and essence of the universe*, *time*, *life*, *death and other metaphysical or existential issues”*.
2. *Personal meaning production–“Ability to create personal intentions*, *purpose and direction in all mental experiences including the capability to establish and implement the purpose of life”*.
3. *Transcendental awareness–“Ability to recognize and understand the superior and transcendental dimensions and aspects of self*, *others and the world in waking life and consciousness”*.
4. *Conscious state expansion–“Ability to enter higher levels of spiritual and beyond consciousness states of mind and exiting from at will”*.

Individuals with higher levels of spiritual intelligence can foresee a more significant purpose in their existence [25]. They are well aware that they are accountable for their actions and responsible towards their surroundings. Particularly in social entrepreneurship, individuals with high spiritual intelligence are more likely to develop a higher degree of accountability to safeguard the interest of society. This is because when they realize that their actions should be “society-specific” and “sustainable”, they extend their participation in social activities [9]. Spiritual intelligence helps individuals view their work in a larger and more meaningful context [25], eliciting the intrinsic valence of their efforts [12]. This is based on the SDT, which posits that by provoking positive feelings nurtured on a prime cause, *e*.*g*., a societal obligation, spiritual intelligence elevates satisfaction and pleasure inherent to the activity. Hence, we expect that spiritual intelligence coupled with intrinsic religiosity exaggerates its impact on intrinsic motivation. However, for individuals with lower spiritual intelligence, the likelihood of cultivating intrinsic religiosity into intrinsic motivation is lesser because their actions are not formed on spiritual values. Hence, their internal drive, *i*.*e*., intrinsic motivation, to execute societal activities is reduced.

Thus,

*H4. Spiritual intelligence moderates the association between intrinsic religiosity and intrinsic motivation such that the association is more potent at higher levels of spiritual intelligence than at low levels*.

The above projections suggest a moderated mediation model [60]. As argued above, spiritual intelligence moderates the association between intrinsic religiosity and intrinsic motivation. Hence, this engagement, in turn, predicts social entrepreneurial intentions. Drawing on the SDT, which purports that people seek actions and experiences to fully internalize their environment into their own interests, which in turns, nurtures meaningful experiences and renders a sense of fulfilment [61]. Through harmonizing ones’ values, actions and interests with those of societal interests and values [12], individuals can cultivate spiritual intelligence into enhanced levels of social entrepreneurial intentions through harnessing intrinsic motivation. We, therefore, propose that spiritual intelligence intervenes in the indirect association between intrinsic religiosity and social entrepreneurial intentions through the mediator effect of intrinsic motivation. Thus,

*H5. Spiritual intelligence moderates the indirect association between intrinsic religiosity and social entrepreneurial intentions through the mediator effect of intrinsic motivation, such that the association is more potent at higher levels of spiritual intelligence than at lower levels*.

## Method

### Sample and procedure

The current study employed a “time-lagged” (*i*.*e*., “three-wave”) research design (with a time interval of eight weeks) to collect data from university students in Punjab, Pakistan. The target respondents are graduating students studying entrepreneurship, economics, management, and computer science programs in their final years. There is an increasing trend of starting own business as an alternative to seeking employment in developing nations [37] to cope with the growing unemployment rate in these countries. Specifically, students pursuing CS and entrepreneurship careers in Pakistan are more inclined to commence their businesses. Furthermore, to meet rising societal pressures and market demands, university students are increasingly focused on initiating social entrepreneurship [3]. Therefore, the target respondents of this study are expected to provide appropriate responses to suffice the purpose of this study. Furthermore, by employing a "time-lagged" research design, the authors have tried to address the issues of biases in estimating parameters, which in the cross-sectional design seems quite problematic [62, 63].

The authors employed a “face-to-face” mode of data collection using a non-probability, “purposive sampling technique”. The reason for utilizing the "purposive sampling technique" is its ability to yield arbitrary responses [64] to achieve the purpose of the study. Ethical approval was obtained from the Research and Ethics Committees under the umbrella of Quality Enhancement Cell (QEC) The University of Faisalabad, Pakistan; Bahria University Islamabad, Pakistan. The ethics committees are operationalized under the QEC of these universities. Further, the authors also received participants’ consent for their voluntary contribution in this survey. The authors contacted the university students to inquire about their consent and availability to participate in the study. The authors distributed questionnaires to the target respondents along with a cover letter specifying the purpose of the study. They were informed that we requested their participation as volunteers across multiple waves, and they could also choose not to participate in the survey at any stage. The cover letter also provided information to generate a key by giving the first letters of their first and last name, ending with the city code. In the first wave, the authors administered 500 questionnaires to obtain responses for intrinsic religiosity, extrinsic religiosity, and spiritual intelligence. Of these, 459 were received. After discarding the 13 incomplete/wrongly filled questionnaires, the remaining 446 questionnaires were distributed in the second wave to seek responses for intrinsic and extrinsic motivation. A total of 429 questionnaires were received. After eight weeks, the authors collected responses for social entrepreneurial intentions.

Finally, the authors consolidated all the responses collected in multiple waves using the key generated by the participants and processed 411 responses using SmartPLS SEM (v 4.0). The participants of this study included 53% of male and 47% of female responses, with a mean age of 29.15 (SD: 5.05). In addition, 30%, 32%, and 38% "undergraduate", "graduate", and "postgraduate" students. Concerning education, the participants were enrolled in entrepreneurship (26%), economics (25%), management (18%), and computer science (31%), respectively.

## Measures

We utilized the established scales for data collection and analysis in this study. To measure religiosity orientation, an 11-item scale (5 statements measuring intrinsic religiosity and 6 statements assessing extrinsic religiosity) was adapted from Allport and Ross [16], modified by Vitell *et al*. [65]. The sample statement included “I try hard to live all my life according to my religious beliefs” (intrinsic religiosity) and “I go to a religious service mostly to spend time with my friends” (extrinsic religiosity). The 9-item scale (5 statements measuring intrinsic motivation and 4 statements assessing extrinsic motivation) to measure intrinsic and extrinsic motivation was adapted from Ryan and Connell [66]. We asked the participants “what motivates you in your current occupation?” and the sample statements included “because I enjoy the work itself” (intrinsic motivation) and “because I want to receive a higher wealth in return in the future”. The 6-item scale to assess social entrepreneurial intention was adapted from Yamini *et al*. [12]. The sample statement included “I have a firm intention to start a social venture someday”. The 24-item scale to measure spiritual intelligence was adapted from King and DeCicco [59]. The sample item included “when I experience a failure, I am still able to find meaning in it”. Three items were eliminated from the scale due to low factor loadings (< 0.70). All the questionnaires were tapped on a 5-point Likert scale ranging from 1 for “strongly disagree” to 5 for “strongly agree” (S1 Appendix).

## Control variables

Following the previous studies, individual demographics, such as age, gender, education, and income, were taken as controlled variables (reported in Table 3).

## Result

### Measurement model

The authors examined the “reflective measurement model” by employing the criteria of “internal consistency” and “convergent and discriminant validity” [67]. For measuring “internal consistency”, the authors employed the metrics of “Cronbach’s alpha”, and “composite reliability” (CR), considering the minimum threshold value of 0.70 [67, 68]. The analysis results are illustrated in Table 1, indicating all the values greater than 0.70. To measure "convergent validity", the authors assessed the "outer loadings" and "average variance extracted" (AVE), considering the minimum threshold value of 0.50 [69, 70]. All the values presented in Table 1 are greater than 0.50, thus validating the convergent validity of the study.

**Table 1:** Validity and reliability for constructs.

	Loadings	AVE	CR	Cronbach’s Alpha
Intrinsic religiosity		0.512	0.898	0.856
IR1	0.743			
IR2	0.753			
IR3	0.723			
IR4	0.712			
IR5	0.642			
Extrinsic religiosity		0.545	0.920	0.871
ER1	0.723			
ER2	0.835			
ER3	0.764			
ER4	0.623			
ER5	0.718			
ER6	0.754			
Intrinsic motivation		0.550	0.876	0.827
IM1	0.717			
IM2	0.802			
IM3	0.628			
IM4	0.746			
IM5	0.791			
Extrinsic motivation		0.581	0.901	0.871
EM1	0.737			
EM2	0.672			
EM3	0.847			
EM4	0.782			
Social entrepreneurial intentions		0.576	0.846	0.780
SEI1	0.726			
SEI2	0.727			
SEI3	0.800			
SEI4	0.798			
SEI5	0.772			
SEI6	0.728			
Spiritual intelligence		0.551	0.870	0.834
SI1	0.723			
SI2	0.654			
SI3	0.743			
SI4	0.653			
SI5	0.864			
SI6	0.775			
SI8	0.734			
SI9	0.623			
SI10	0.775			
SI12	0.723			
SI13	0.786			
SI14	0.772			
SI15	0.827			
SI16	0.701			
SI17	0.681			
SI18	0.718			
SI19	0.742			
SI20	0.728			
SI22	0.773			
SI23	0.732			
SI24	0.773			

*Notes*. IR: intrinsic religiosity; ER: extrinsic religiosity; IM: intrinsic motivation; EM: extrinsic motivation; SEI: social entrepreneurial intentions; SI: spiritual intelligence

Moreover, the authors also examined the “discriminant validity” using the criteria of “Fornell-Larcker” and “heterotrait-monotrait” (HTMT) ratio. The Fornell-Larcker measures the square root of AVE in the construct correlation matrix and indicates that all the square root values of AVE are higher for their construct than the related inter-construct correlation [67] (Table 2). Besides, the authors employed a "bias-corrected and accelerated" (BCa) bootstrapping approach with a resample of 5,000 using a one-tailed *t*-test at a 90% significance level to yield an error probability of 5%. [71]. The HTMT ratio presented in Table 3 confirms the discriminant validity of the study as all the values should be lesser than the maximum threshold, *i*.*e*., HTMT_.85_.

**Table 2:** Fornell-Larcker criterion.

	IR	ER	IM	EM	SEI	SI
IR	0.715					
ER	0.442	0.738				
IM	0.545	0.534	0.743			
EM	0.323	0.452	0.432	0.762		
SEI	0.242	0.432	0.343	0.534	0.759	
SI	0.520	0.524	0.334	0.423	0.643	0.742

*Notes*: IR: intrinsic religiosity; ER: extrinsic religiosity; IM: intrinsic motivation; EM: extrinsic motivation; SEI: social entrepreneurial intentions; SI: spiritual intelligence

**Table 3:** HTMT criterion.

	IR	ER	IM	EM	SEI	SI
IR						
ER	0.700 CI._0.900_ [0.645;0.761]					
IM	0.617 CI._0.900_ [0.558;0.671]	0.623 CI._0.900_ [0.548;0.680]				
EM	0.600 CI._0.900_ [0.533;0.664]	0.729 CI._0.900_ [0.667;0.777]	0.823 CI._0.900_ [0.757;0.884]			
SEI	0.690 CI._0.900_ [0.631;0.752]	0.676 CI._0.900_ [0.612;0.742]	0.790 CI._0.900_ [0.710;0.868]	0.625 CI._0.900_ [0.578;0.670]		
SI	0.472 CI._0.900_ [0.412;0.534]	0.772 CI._0.900_ [0.690;0.834]	0.636 CI._0.900_ [0.578;0.702]	0.526 CI._0.900_ [0.442;0.591]	0.753 CI._0.900_ [0.681;0.820]	

*Notes*. IR: intrinsic religiosity; ER: extrinsic religiosity; IM: intrinsic motivation; EM: extrinsic motivation; SEI: social entrepreneurial intentions; SI: spiritual intelligence; CI: bootstrapping 90% confidence intervals (n = 5,000) (one-tailed)

### Structural model

After validating the measurement model, the authors employed the PLS algorithm to yield the "path coefficients" (*β*), “coefficient of determination” (*R*^*2*^), “predictive relevance” (*Q*^*2*^), and “effect size” (*f*^*2*^). Furthermore, to obtain the corresponding *t*- and *p*-values, the authors ran BCa bootstrapping (5,000 resamples) at a 95% significance level [67]. Results of the direct effects are presented in Table 4, supporting the proposed hypotheses. The analysis indicates that religiosity, *i*.*e*., intrinsic religiosity has a significant positive association with intrinsic motivation (*β* = 0.480, *t* = 10.061, *p* = 0.000, *f*^*2*^ = 0.362) (supporting *H1a*), and extrinsic religiosity has a significant positive association with extrinsic motivation (*β* = 0.360, *t* = 8.855, *p* = 0.000, *f*^*2*^ = 0.394) (supporting *H1b*). Further, intrinsic motivation has a significant positive association with social entrepreneurial intentions (*β* = 0.540, *t* = 6.784, *p* = 0.000, *f*^*2*^ = 0.198) (supporting *H2a*), and extrinsic motivation has a significant positive association with social entrepreneurial intentions (*β* = 0.474, *t* = 9.782, *p* = 0.000, *f*^*2*^ = 0.248) (supporting *H2b*).

**Table 4:** Effects on endogenous variables.

Hypotheses	*β*	CI (5%, 95%)	SE	*t*-value	*p*-value	Decision	*f* ^*2*^	*R* ^*2*^	*Q* ^*2*^
Age^1^	0.048(*n*.*s*.)	(-0.039, 0.089)	0.021	0.391	0.342				
Gender^2^	0.087(*n*.*s*.)	(-0.028, 0.154)	0.012	0.663	0.531				
Education^3^	0.053(*n*.*s*.)	(-0.031, 0.084)	0.028	0.347	0.444				
Income^4^	0.011(*n*.*s*.)	(-0.042, 0.089)	0.031	1.11	0.280				
*H1a* IR → IM	0.480***	(0.390, 0.572)	0.048	10.061	0.000	Supported	0.362	0.562	0.332
*H1b* ER → EM	0.360***	(0.298, 0.446)	0.064	8.855	0.000	Supported	0.394	0.482	0.464
*H2a* IM → SEI	0.540***	(0.468, 0.610)	0.052	6.784	0.000	Supported	0.198	0.612	0.354
*H2b* EM → SEI	0.474***	(0.410, 0.542)	0.061	9.782	0.000	Supported	0.248		
*H4* IR x SI → IM	0.412***	(0.334, 0.478)	0.068	11.324	0.000	Supported	0.281		
*H5* IR x SI → SEI	0.394***	(0.322, 0.460)	0.040	8.420	0.000	Supported	0.217		

*Notes*. IR: intrinsic religiosity; ER: extrinsic religiosity; IM: intrinsic motivation; EM: extrinsic motivation; SEI: social entrepreneurial intentions; SI: spiritual intelligence; ***significance p < 0.05 (1.96); ^1,2,3,4^ = control variables

Moreover, the study also projected the mediating roles of intrinsic motivation and extrinsic motivation in the underlying associations. The authors used Zhao *et al*.*’s* [72] mediation approach to examine the mediator effects. The authors assessed the BCa on 5,000 resamples to yield indirect effects [67]. Results of this analysis are presented in Table 5 which shows that both the direct effects for intrinsic religiosity → social entrepreneurial intentions (*β* = 0.390, CI = 0.332, 0.470) and extrinsic religiosity → social entrepreneurial intentions (*β* = 0.326, CI = 0.256, 0.394) are significant. In addition, both the indirect effects for intrinsic religiosity → intrinsic motivation → social entrepreneurial intentions (*β* = 0.284, CI = 0.226, 0.348) and extrinsic religiosity → extrinsic motivation → social entrepreneurial intentions (*β* = 0.312, CI = 0.245, 0.381) are significant, indicating “complementary mediation” [67]. The authors also assessed the "variance accounted for" (VAF) to determine the mediation analysis. The VAF values for *H3a* (42.13%) and *H3b* (48.90%) illustrated in Table 5 confirm the significant mediation of intrinsic and extrinsic motivation between intrinsic and extrinsic religiosity and social entrepreneurial intentions.

**Table 5:** Summary of mediating effect tests.

	Path	*t*-value	95% BCCI		Path	*t*-value	95% BCCI	Decision	VAF
Direct effect				Indirect effect					
IR → SEI	0.390	6.238	(0.332, 0.470)	IR → IM → SEI	0.284	7.414	(0.226, 0.348)	Supported	42.13%
ER → SEI	0.326	4.252	(0.256, 0.394)	ER → EM → SEI	0.312	5.350	(0.245, 0.381)	Supported	48.90%

*Notes*. IR: intrinsic religiosity; ER: extrinsic religiosity; IM: intrinsic motivation; EM: extrinsic motivation; SEI: social entrepreneurial intentions; SI: spiritual intelligence; VAF: variance accounted for (indirect effect / total effect*) *total effect: direct effect + indirect effect)

To measure the moderation effect of spiritual intelligence, the authors used a "two-stage approach" to yield the effect sizes and CIs in line with the recommendations of [67]. Further, Henseler and Fassott [73] argue that the “two-stage approach” has superior statistical power than the “product indicator” and “orthogonal” approaches. The interaction effects are presented in Table 4. The analysis confirms the interaction effects between intrinsic religiosity*spiritual intelligence on intrinsic motivation (*β* = 0.412, CI = 0.334, 0.478) and intrinsic religiosity*spiritual intelligence on social entrepreneurial intentions through the mediator effect of intrinsic motivation (*β* = 0.394, CI = 0.322, 0.460), are significant and support *H4* and *H5*. Besides, *f*^*2*^ indicates the medium effect sizes.

**Fig 2:**
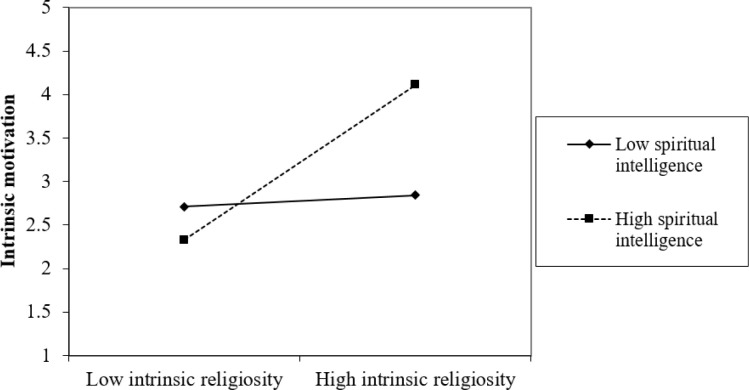
Interaction effect of intrinsic religiosity and spiritual intelligence on intrinsic motivation.

**Fig 3:**
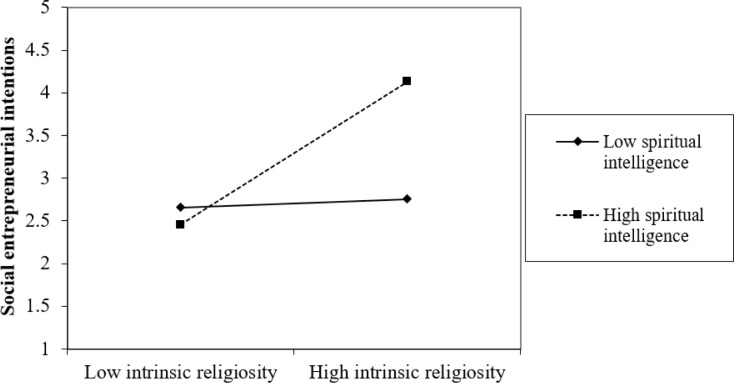
Interaction effect of intrinsic religiosity and spiritual intelligence on social entrepreneurial intentions.

The authors also assessed the "simple slope analysis", as Dawson [74] recommended. The results of this analysis are presented in Figs 2 and 3. The slopes indicate that at high levels of spiritual intelligence, the associations between (1) intrinsic religiosity and intrinsic motivation and (2) intrinsic religiosity and social entrepreneurial intentions, mediated by intrinsic motivation, are more pronounced than at low levels of spiritual intelligence, rendering support to *H4* and *H5*.

Furthermore, the authors also evaluated the “goodness-of-fit” index (GFI) by employing the Tenenhaus *et al*. [75] diagnostic tool. GFI refers to "the geometric mean of the average communality and average *R*^*2*^”. The results of this analysis are shown in Table 6. The GFI value of 0.551 is greater than the cutoff value of 0.36 to ensure the large effect size of *R*^*2*^, ensuring a good model fit [76].

**Table 6:** Goodness-of-Fit Index (GFI).

Constructs	AVE	*R* ^*2*^
IR	0.512	
ER	0.545	
IM	0.550	0.562
EM	0.581	0.482
SEI	0.576	0.612
SI	0.551	
Average scores	0.552	0.552
(*GFI* = $\sqrt{\bar{AVE}\times \bar{R^{2}}}$)	0.551	

*Notes*. AVE: average variance extracted; IR: intrinsic religiosity; ER: extrinsic religiosity; IM: intrinsic motivation; EM: extrinsic motivation; SEI: social entrepreneurial intentions; SI: spiritual intelligence

Lastly, for estimating the “predictive relevance”, the authors utilized Stone-Geisser’s *Q*^*2*^ with an “omission distance” of 7 to obtain the “cross-validated redundancy” (*Q*^*2*^), and the values above 0 ensure the predictive relevance of the hypothesized model.

## Discussion and conclusion

Despite the numerous discussion that religiosity and spirituality can contribute to students’ intentions to start their own business [2, 14, 37], the nature of the relationship between religiosity, spiritual intelligence, and social entrepreneurial intentions has been neglected, which is a crucial omission to be addressed. By considering motivation (*i*.*e*., intrinsic motivation and extrinsic motivation) as a mediator and spiritual intelligence as a moderator, we investigated internal mechanisms behind the religiosity (*i*.*e*., intrinsic religiosity and extrinsic religiosity) and social entrepreneurial intentions link. The study employs a “time-lagged” research design to collect data from university students in Pakistan and analyzes using SmartPLS (v 4.0). Our findings present unique and novel insights and reveal that religiosity influences social entrepreneurial intentions through two paths: intrinsic religiosity, which draws on the inherent religious values to affect social entrepreneurial intentions through intrinsic motivation, and extrinsic religiosity, which emphasizes external benefits innate to religious practices impact social entrepreneurial intentions through extrinsic motivation. In addition, our results indicate that spiritual intelligence is an intervening factor that intertwines values and intelligence and underpins the underlying association between intrinsic religiosity and social entrepreneurial intentions through the mediating role of intrinsic motivation.

### Theoretical implications

We expect several novel and meaningful theoretical implications from this study. First, despite popular press articles emphasising the importance of social entrepreneurial intentions as crucial in translating into social entrepreneurial behaviors [11], entrepreneurship research is striving to investigate factors that might elevate university students’ social entrepreneurial intentions [37]. In this regard, growing empirical research has employed several theories to elucidate factors that shape students’ entrepreneurial intentions. For instance, entrepreneurial event and cultural values theory [76], person-entrepreneurship fit theory [77], theory of social cognitive [14], resource-based theory [78], theory of planned behavior [50], and theory of reasoned action [14], there are repeated calls to investigate the boundary conditions of students’ social entrepreneurial intentions [1, 5, 11]. Further, most prior research exploring the antecedents of social entrepreneurial intentions has examined contextual factors [54]. Moreover, further attempts have been made to examine individual characteristics, *such as* entrepreneurial personality profiles, in shaping social entrepreneurial intentions [13]. However, the path from religiosity to social entrepreneurial intentions through motivation has never been tested earlier. Hence, our study advances existing research on the antecedents of social entrepreneurial intentions by proposing that individual’s religious orientations, *i*.*e*., intrinsic religiosity and extrinsic religiosity, play vital role in shaping university students’ social entrepreneurial intentions. In addition, by examining the relationship between religiosity and social entrepreneurial intentions through dual paths, our findings advance the study of McIntyre *et al*. [36], who examined the relationship between religiosity and social entrepreneurial intentions.

Second, research studies are on the rise exploring the consequences of individuals’ religious orientations in the work context [35] and entrepreneurship domains [38]. However, findings from prior studies reveal that the relationship between religiosity and entrepreneurship is not always unidirectional [37] or, in some cases, is nonexistent [39]. Moreover, extant preliminary research has observed a positive association between intrinsic religiosity and entrepreneurship, whereas the relationship concerning extrinsic religiosity has remained uncertain [46]. Thus, our study furthers the current debate on the link between religiosity orientations and entrepreneurship by projecting the positive impacts of intrinsic and extrinsic religiosity on social entrepreneurial intentions. Moreover, we anticipate the implications of our findings beyond the entrepreneurship domain to the work contexts. Drawing on the SDT, both types of religiosity are crucial for individuals. They may complement each other to predict a wide range of work behaviors *such as* organizational citizenship behaviors [79], work ethics [46], and prosocial and altruistic behaviors [19], among others.

Moreover, investigating motivation as a causal mechanism in the indirect relationship between religiosity and social entrepreneurial intentions turns out to be our third contribution. To the best of the authors’ knowledge, no prior studies have tested these relationships. Furthermore, the relationship between intrinsic religiosity and social entrepreneurial intentions, mediated by intrinsic motivation, is plausible and verified in several studies in different contexts and combinations [46]. However, the significant mediating role of extrinsic motivation between extrinsic motivation and social entrepreneurial intentions is epoch-making that furthers the implications of SDT in occupational settings [47].

Last but not least, our study advances the extant literature on entrepreneurship by expanding the boundary conditions of religiosity and social entrepreneurial intentions under which the relationship is more or less likely to pronounce. We answer the repeated calls of Cardella *et al*.; García-Jurado *et al*.; Hossain *et al*. [1, 5, 11], and project the boundary effects of spiritual intelligence in the direct relationship between intrinsic religiosity and intrinsic motivation, and the indirect relationship between intrinsic religiosity and social entrepreneurial intentions through the mediator effect of intrinsic motivation. Our findings reveal that the relationship between intrinsic religiosity and social entrepreneurial intentions, mediated by intrinsic motivation, is stronger are higher levels of spiritual intelligence and vice versa. Our findings are in congruence with the previous research on the association between spiritual intelligence, self-efficacy, and entrepreneurial passion [25]; thereby expanding the theoretical and empirical implications of the spiritual intelligence literature.

### Practical implications

The present study offers numerous important insights for practical implications. First, given the crucial role of social entrepreneurship in contributing to the country’s economy in multiple ways (*e*.*g*., coping with escalating unemployment, elevating economic and sustainable development, contributing to societal needs and issues, offering opportunities to opt alternate career choices for students, etc.), research studies unearthing the boundary conditions of the phenomenon are of supreme importance. In other words, social entrepreneurs establish an enterprise with a social mission to cope with increasing societal issues and offer products/services through sustainable business practices. For this sake, government and policymakers should encourage university students to establish social enterprises that serve society at large. Several prior studies have endorsed that governmental and institutional support (*e*.*g*., accessible financing and consultancy, business incubations, etc.) facilitate the culmination of entrepreneurial intentions into entrepreneurial behaviors.

Second, our study highlights the significance of religiosity, *i*.*e*., intrinsic religiosity and extrinsic religiosity, in determining university students’ social entrepreneurial intentions. Based on these suggested associations, consultants, teachers, and policymakers may have appropriate tools and tips when working with individuals who intend to commence social entrepreneurship. In this regard, micro and macro policies, training to nurture entrepreneurial mindset and skill set, and teaching curriculums may be more effective in facilitating students to foster social entrepreneurial intentions. For instance, Toledano [15] researched the influence of religious parables as narratives on the ethical discussion in social entrepreneurship courses. This is one of the examples through which entrepreneurship teachers can nurture social entrepreneurial intentions by inculcating religious beliefs and values. Moreover, teachers should act as role models and practice religion in their day-to-day life experiences so that students can take inspiration from their teachers and cultivate religious perspectives in their lifestyles. Prior studies suggest that 80% of individuals worldwide recognize religion as a critical element of their life [80].

In addition, the study finds that both types of religious orientations, *i*.*e*., intrinsic religiosity and extrinsic religiosity, are essential in transforming social entrepreneurial intentions. The SDT supports our theoretical deduction and postulates that intrinsic motivation mediates the link between intrinsic religiosity and social entrepreneurial intentions, while extrinsic religiosity mediates the association between extrinsic religiosity and social entrepreneurial intentions. Drawing on the SDT, we recommend that institutions should nurture motivation in their students by providing environments that fulfil students’ “autonomy”, “relatedness”, and “competence” needs. Universities should organize inter-and intra-university business ideas competitions and business galas so that students should be intrinsically and extrinsically motivated to participate in such events. Literature suggests that such events encourage students to establish their businesses and facilitate the transformation of ideas into reality by seeking experts’ experiences and suggestions.

Last but not least, our study finds that spiritual intelligence significantly moderates the direct relationship between intrinsic religiosity and intrinsic motivation and the indirect relationship between intrinsic religiosity and social entrepreneurial intentions mediated by intrinsic motivation. Universities should play a critical role in defining and instilling a meaningful purpose in life for their students. Universities should offer a platform to their students through which they can take part in social activities. Numerous societies should be established in the universities (*e*.*g*., welfare society, community services, women’s and children’s health and protection, etc.), and students should be encouraged to take an active part and give their best to promote social interest at large. Our findings suggest that individuals with meaning in life possess higher levels of spiritual intelligence, which ultimately underpins the association between intrinsic religiosity and social entrepreneurial intentions mediated by intrinsic motivation.

### Limitations of the study

Although the current study presents unique and meaningful insights, our findings should be studied with limitations. First, the study employs a time-lagged research design to minimize the issues of biases in the parameter estimation [62, 63]. Despite that, all the study variables are not tapped at all periods; therefore, future studies should utilize a longitudinal design to test the hypothesized model. Second, the study outstretches the boundary conditions of the religiosity (*i*.*e*., intrinsic religiosity and extrinsic religiosity) and social entrepreneurial intentions nexus by proposing motivation (*i*.*e*., intrinsic motivation and extrinsic motivation) as a mediating variable. The study finds that both types of motivation, *i*.*e*., intrinsic motivation and extrinsic motivation, *partially* mediate the associations between intrinsic religiosity and extrinsic religiosity and social entrepreneurial intentions. This indicates that the association might also be influenced by some other contingent factors not tested in this study. Therefore, future studies may investigate other mediating variables, such as entrepreneurs’ social and moral identification [81] and intrinsic and extrinsic goals [51], to explore the underlying causal mechanism. Third, future studies should extend the boundary conditions of the religiosity–social entrepreneurial intentions linkage by investigating other moderators to understand under what conditions the associations are more or less likely to be pronounced. Further, we invite future studies to examine the boundary effects of the demographic profiles of the respondents as these may have influence on the underlying linkages. Last but not least, the findings of this study reflect insights of university students in a non-Western cultural context, characterized by high collectivism; therefore, the findings of this study should not be generalized to Western countries, *i*.*e*., individualistic culture. Therefore, we invite future studies to test the hypothesized relationships in Western contexts.

## Supporting information

S1 Appendix Questionnaire. (DOCX)
