# Supplementary material for: “Entrepreneurship” from the lens of enlightenment: Impacts of religiosity and spiritual intelligence on social entrepreneurial intentions
Source: PLoS One. 2023 Oct 10;18(10):e0285140. doi: 10.1371/journal.pone.0285140 (PMC10564168; doi:10.1371/journal.pone.0285140)
Supplement: S1 Appendix — (DOCX) [file pone.0285140.s001.docx]

**Appendix I: Questionnaire**

**Intrinsic religiosity**

1. I enjoy reading about my religion.
2. My whole approach to life is based on religion.
3. It is important to me to spend time in private thought and prayer.
4. I have often had a strong sense of God’s presence.
5. I try hard to live all my life according to my religious beliefs.

**Extrinsic religiosity**

1. I pray mainly to gain relief and protection.
2. What religion offers me most is comfort in times of trouble and sorrow.
3. Prayer is for peace and happiness.
4. I go to a religious service mostly to spend time with my friends.
5. I go to religious services because I enjoy seeing people I know there.
6. I go to religious services because it helps me to make friends.

**Intrinsic motivation**

What motivates you in your current occupation (work/study, etc.)?

1. because my work gives me a feeling of personal accomplishment.
2. because I enjoy the work itself
3. because the work I do is important.
4. because it’s fun
5. because I find the work engaging

**Extrinsic motivation**

What motivates you in your current occupation (work/study, etc.)?

1. because I want to receive a higher wealth in return in the future.
2. because I want to have a better standard of living
3. because I want a higher income
4. because I want to have a better social status

**Social entrepreneurial intentions**

1. I am ready to do anything to be a social entrepreneur that helps society.
2. My professional goal is to be a social entrepreneur.
3. I will make every effort to start and run my own venture that helps society.
4. I am very determined to create a venture that helps society in the future.
5. I have very seriously thought of starting a firm that helps society in some way.
6. I have the firm intention to start a social venture someday.

**Spiritual intelligence**

1. I have often questioned or pondered the nature of reality.
2. I recognize aspects of myself that are deeper than my physical body.
3. I have spent time contemplating the purpose or reason for my existence.
4. I am able to enter higher states of consciousness or awareness.
5. I am able to deeply contemplate what happens after death.
6. It is difficult for me to sense anything other than the physical and material. (R)
7. My ability to find meaning and purpose in life helps me adapt to stressful situations.
8. I can control when I enter higher states of consciousness or awareness.
9. I have developed my own theories about such things as life, death, reality, and existence.
10. I am aware of a deeper connection between myself and other people.
11. I am able to define a purpose or reason for my life.
12. I am able to move freely between levels of consciousness or awareness.
13. I frequently contemplate the meaning of events in my life.
14. I define myself by my deeper, non-physical self.
15. When I experience a failure, I am still able to find meaning in it.
16. I often see issues and choices more clearly while in higher states of consciousness/ awareness.
17. I have often contemplated the relationship between human beings and the rest of the universe.
18. I am highly aware of the nonmaterial aspects of life.
19. I am able to make decisions according to my purpose in life.
20. I recognize qualities in people which are more meaningful than their body, personality, or emotions.
21. I have deeply contemplated whether or not there is some greater power or force (e.g., god, goddess, divine being, higher energy, etc.).
22. Recognizing the nonmaterial aspects of life helps me feel centered.
23. I am able to find meaning and purpose in my everyday experiences.
24. I have developed my own techniques for entering higher states of consciousness or awareness.
